# Exploration of the distribution of intestinal bacteria in mice under normal and intestinal leakage (IBD) conditions

**DOI:** 10.1099/jmm.0.002054

**Published:** 2025-09-03

**Authors:** Jinhuan Niu, Wei Shi, Ruijia Zhu, Xiaoyue Hu, Meiliang Pan, Fan Zhang, Weiping Fan

**Affiliations:** 1 Department of Microbiology and Immunology, Shanxi Medical University, Shanxi, PR China; 2 Shanxi Provincial People’s Hospital, Shanxi, PR China

**Keywords:** bacterial translocation, GFP-labelled *Escherichia coli*, intestinal leakage

## Abstract

**Introduction.** The distribution of micro-organisms in healthy organisms remains a subject of debate. Emerging evidence revealed the colonization of microbial communities in multiple anatomical sites previously considered sterile under homeostatic conditions. However, the mechanistic relationship between compromised intestinal epithelial barrier integrity and subsequent translocation of gut-resident bacteria into systemic circulation has yet to be comprehensively elucidated.

**Hypothesis/Gap Statement.** Under intestinal leakage, gut micro-organisms can break through the intestinal barrier and then translocate to other organs.

**Aim.** This study investigates the distribution of micro-organisms in healthy organisms to determine whether gut bacteria translocate to sterile organs only under the condition of intestinal leakage using GFP-labelled *Escherichia coli* (GFP-*E. coli*) tracing.

**Methodology.** Female C57BL/6 mice (5 weeks old) were administered either a glacial acetic acid enema [inflammatory bowel disease (IBD) group] or a sterile normal saline enema [normal control (NC) group]. All mice were subsequently gavaged with GFP-*E. coli*. HE staining and Alcian blue staining were performed to evaluate the colon injury. The expression levels of intestinal tight junction proteins (ZO-1 and occludin) were tested by reverse transcription quantitative PCR and immunofluorescence staining. The distribution of GFP-*E. coli* in multiple organs was assessed through bacterial culture, confocal microscopy and PCR.

**Results.** In the IBD mice, mucopolysaccharide accumulation levels (*P*<0.01) and tight junction proteins ZO-1 (*P*<0.001) and occludin (*P*<0.01) in the colon were significantly decreased compared with the NC group. Bacterial culture showed that there was no GFP-*E. coli* in the blood, heart, liver, spleen, lungs, kidneys or oviducts of normal mice, while the number of GFP-*E. coli* colonies in the blood (219 c.f.u. ml^−1^), liver (2.39×10^5^ c.f.u. ml^−1^) and lungs (2.50×10^8^ c.f.u. ml^−1^) of the IBD mice was significantly higher than that of the NC group. The confocal microscopy and PCR results also showed that the number of GFP-*E. coli* in the liver and lungs of the IBD group was significantly higher than that of the NC group (*P*<0.001).

**Conclusion.** Healthy mice maintain a sterile microenvironment in the blood, heart, liver, spleen, lungs, kidneys and oviducts. However, compromised intestinal barrier integrity facilitates microbial translocation from the intestinal lumen into the blood, liver and lungs. This study advances our understanding of endogenous infections caused by IBD, demonstrating the crucial role of intestinal permeability in bacterial infections.

## Data Summary

The authors confirm that all supporting data and protocols have been provided in the article.

## Introduction

It is generally accepted that commensal microbiota predominantly colonize the distal ends of the lumens that communicate with the outside, such as the oral cavity, the intestine, the anus of the digestive tract, the nasopharynx of the respiratory tract and certain parts of the urogenital tract [1]. In contrast, the internal solid organs (heart, liver, spleen, lungs and kidneys) are conventionally considered to be microbially sterile under homeostatic conditions. However, recent evidence has demonstrated the presence of microbial communities in traditionally presumed sterile human tissues, including liver, uterus and placenta [2]. We assume that the key of the dispute lies in the permeability of the intestinal wall. That is, under conditions of intestinal leakage, gut micro-organisms can break through the intestinal barrier, enter the blood through the portal vein and then translocate to organs throughout the body [3].

Inflammatory bowel disease (IBD) serves as a paradigmatic model for studying microbial translocation due to its characteristic disruption of intestinal barrier integrity and induction of increased intestinal permeability [4]. The species and distribution of gut microbiota in IBD patients are significantly altered, indicating a state of dysbiosis [5]. *Escherichia coli* is an important member of the gut microbiota, and its changes in the IBD environment may have a crucial impact on the progression of various diseases [6]. GFP-labelled *E. coli* (GFP-*E. coli*) serves as a tracking tool for bacterial colonization, translocation and host–microbe interactions. In this study, we used GFP-*E. coli* tracing to analyse whether gut bacteria translocate to sterile tissues and organs only under the condition of intestinal leakage.

## Methods

### Primers

The required primers were designed using the Primer 5.0 software and synthesized by Shanghai Sangon Biotech (Shanghai) Co., Ltd. The primer sequences (Table 1)were as follows.

**Table 1.** Primer sequence

Gene name	Primer direction	Sequence (5′–3′)
*β*-Actin	Forward reverse	CACTGTCGAGTCGCGTCC TCATCCATGGCGAACTGGTG
ZO-1	Forward reverse	GCGAACAGAAGGAGCGAGAAGAG GCTTTGCGGGCTGACTGGAG
Occludin	Forward reverse	TGGACTTGGAGGCGGCTATGG AGGGAAGCGATGAAGCAGAAGGC
*E. coli*-16S	Forward reverse	CAACGAGCGCAACCCTTATC CGGACTACGACGCACTTTATGAG

### Animals and IBD mouse modelling

All animal experiments were approved by the Ethics Committee of Shanxi Medical University (Shanxi, China). Twelve female C57BL/6 mice [5 weeks old, SYXK (Jing) 2022-0003] were purchased from Beijing Huafukang Biotechnology Co., Ltd. (Beijing, China) and randomly divided into the IBD group (*n*=6) and the NC (normal control) group (*n*=6). The mice were housed in an animal facility under standard SPF conditions at 24 °C on a 12 h light/dark schedule, with free access to water and food. For colitis induction, all mice were fasted for 12 h prior to chemical intervention. The IBD group received intracolonic administration of 1 ml of 5% (v/v) glacial acetic acid (Sigma-Aldrich, USA), while the NC group received equivalent volumes of sterile saline. This procedure was repeated after 36 h to establish chronic mucosal injury. Mice with bloody faeces are a successful IBD model.

### Culture of GFP-*E. coli*

A single colony of GFP-*E. coli* (P33422, Takara Company, China) was picked and inoculated into LB broth supplemented with 100 µg ml^−1^ ampicillin. The culture was incubated in an orbital shaker (37 °C, 200 r.p.m.), until mid-log phase (OD_600_ 0.6–0.8). Then, 1 ml IPTG solution (1 mol l^−1^) was added to the liquid medium, and cultures were continued for 20 h at 18 °C, 200 r.p.m.

### GFP-*E. coli* gavage and tissue collection

Following successful model establishment, 2 ml of ampicillin-resistant GFP-*E. coli* suspension (5×10⁸ c.f.u. ml^−1^) was orally administered to both NC and IBD mice. Twelve hours later, the gavage procedure was repeated. At 24 h post-initial gavage, mice were anesthetized with isoflurane gas (3%–4%) and sacrificed by cervical dislocation.

Blood, colon, heart, liver, spleen, lungs, kidneys and oviduct samples were aseptically collected and either stored at −80 °C.

### Histological morphology of the colon in mice

After being fixed in 10% formalin solution for 24 h, the colon tissues were paraffin-embedded, sectioned (3 µm) and stained using haematoxylin and eosin (Minibio, China). The sections were stained with haematoxylin solution for 10 min and then rinsed with tap water. Then, the samples were disposed of with a differentiating solution for 30 s and soaked in water for 15 min. Next, the sections were stained with eosin solution for 1 min, washed with water, dehydrated and cleared with gradient ethanol and xylene and finally sealed with neutral gum. The morphology of tissue was observed under a microscope (Carl Zeiss AG, Germany).

### Detection of mucin by Alcian blue staining

Colon tissues were fixed as before, and 5-µm-thick paraffin-embedded sections were cut and stained with Alcian blue (Solarbio, China). The sections were dewaxed and re-hydrated and rinsed with PBS. Then, they were immersed in Alcian acidified working solution for 5 min in the dark, and then in the staining solution for 50 min. After staining with nuclear fast red, the sections were dehydrated and cleared through a gradient of ethanol, xylene and sealed with neutral gum. The mucin was observed under the microscope and quantified by ImageJ software.

### Detection of ZO-1 and occludin mRNA expression levels by qRT-PCR

Total RNA was extracted from the mouse colon tissue with the RNAkey Reagent (Seven Biotech, China) and used for cDNA synthesis. Mouse colon tissue was disrupted by ultrasound, and 0.4 ml of chloroform was added, mixed by vortex and followed by centrifugation at 12,000 r.p.m. for 15 min at 4 °C. The supernatant was removed and precipitated with isopropanol, washed and centrifuged. Finally, Diethyl pyrocarbonate-treated water was added to dissolve the precipitate. The concentration of RNA was determined by a nucleic acid detector. For quantitative reverse transcription PCR (qRT-PCR) analysis, cDNA was synthesized using the Prime Script^™^ RT Reagent Kit with gDNA Eraser (Takara, Japan), and PCR was performed by the TB Green-based assay using synthesized cDNA as the template on the QuantStudio^™^ 3 system (Thermo Fisher Scientific, USA). The reaction systems of qRT-PCR contained 10 µl TB Green Premix Ex Taq II, 0.8 µl each of forward and reverse primers (10 µM), 0.4 µl ROX Reference Dye (50×), 2 µl diluted cDNA and nuclease-free distilled water to a final volume of 20 µl. The thermal cycling conditions were as follows: 95 °C for 10 s followed by 40 cycles of qRT-PCR (95 °C for 5 s, 60 °C for 30 s and 72 °C for 30 s). All qRT-PCR assays included six biological replicates (*n*=6) and three technical replicates (three wells per sample). Relative mRNA levels were normalized against *β*-actin mRNA levels, and the method of 2^-ΔΔ^Ct was adopted to calculate the relative expression of occludin and ZO-1.

### Detection of the expression of ZO-1 and occludin by immunofluorescence staining

The colon tissues were fixed in 10% formaldehyde solution for 48 h, embedded with paraffin and cut into 4-µm-thick sections. For antigen retrieval, the sections were immersed in sodium citrate buffer and microwaved at high power for 10 min, followed by cooling to room temperature. After rinsing with PBS, the sections were blocked with 5% BSA solution for 1 h at room temperature. The tissue sections were then incubated overnight at 4 °C with primary antibodies, including anti-ZO-1 (rabbit anti-mouse, Abcam, UK) and anti-occludin (rabbit anti-mouse, Abcam, UK). Following primary antibody incubation, the sections were treated with an Alexa Fluor 555-labelled secondary antibody CY3 (goat anti-rabbit, BBI, UK) and incubated at room temperature in the dark for 1 h. Finally, the stained sections were visualized using a fluorescence microscope (Carl Zeiss AG, Germany), and the fluorescence intensity was quantified using ImageJ software.

### Detection of GFP-*E. coli* in various tissues of mice

Blood, heart, liver, spleen, lungs, kidneys and oviducts were collected from the mice. The heart, liver, spleen, lungs, kidneys and oviduct tissues were aseptically homogenized in 10 ml of buffer, with the heart, liver, spleen, lungs and kidneys processed at 40 mg each, and the oviducts at 2 mg. The liver tissue homogenate was serially diluted tenfold four times, while the lung tissue homogenate was serially diluted tenfold eight times. Subsequently, 1 ml of blood and 1 ml of each tissue homogenate were plated onto ampicillin-containing Petri dishes, incubated at 37 °C for 24 h and used to quantify GFP-*E. coli*.

### Detection of the distribution of GFP-*E. coli* in liver and lung tissues by confocal microscopy

Frozen liver and lung tissues were embedded in OCT compound and sectioned into 10 µm slices. The sections were stained with DAPI by dropwise application and incubated at room temperature in the dark for 10 min. After rinsing with PBS, the sections were mounted with glycerol gelatin and visualized using a confocal microscope (Olympus, Japan) at a magnification of ×1,000. The distribution of GFP-*E. coli* was quantified using ImageJ software.

### PCR detection of *E. coli* in mouse liver and lung tissues

Total RNA was extracted from mouse liver and lung tissues using the RNAkey Reagent (Seven Biotech, China). cDNA was synthesized from the extracted RNA using the PrimeScript^™^ RT Reagent Kit with gDNA Eraser (TaKaRa, Japan), following the manufacturer’s protocol. PCR amplification was performed using the synthesized cDNA as a template on a Veriti 96-Well Thermal Cycler system (Thermo Fisher Scientific, USA). The PCR reaction mixture consisted of the following components: 2.5 µl of 10× PCR buffer, 5 µl of template DNA, 0.5 µl of forward primer (10 µM), 0.5 µl of reverse primer (10 µM) (Table 1), 2.5 µl of dNTPs, 0.25 µl of Taq DNA polymerase and nuclease-free distilled water to a final volume of 25 µl. The PCR conditions included an initial denaturation step at 95 °C for 5 min, followed by 40 cycles of amplification (95 °C for 10 s, 60 °C for 34 s and 72 °C for 16 s). After PCR amplification, the products were separated by electrophoresis on 1% agarose gel.

### Statistics

Statistical analysis comparing NC and IBD groups was carried out using the unpaired t-test using GraphPad Prism Version 8.4.1. *P*-values lower than 0.05 were considered significant.

## Results

### Impaired morphological structure of the colon in IBD mice

**Fig. 1.**
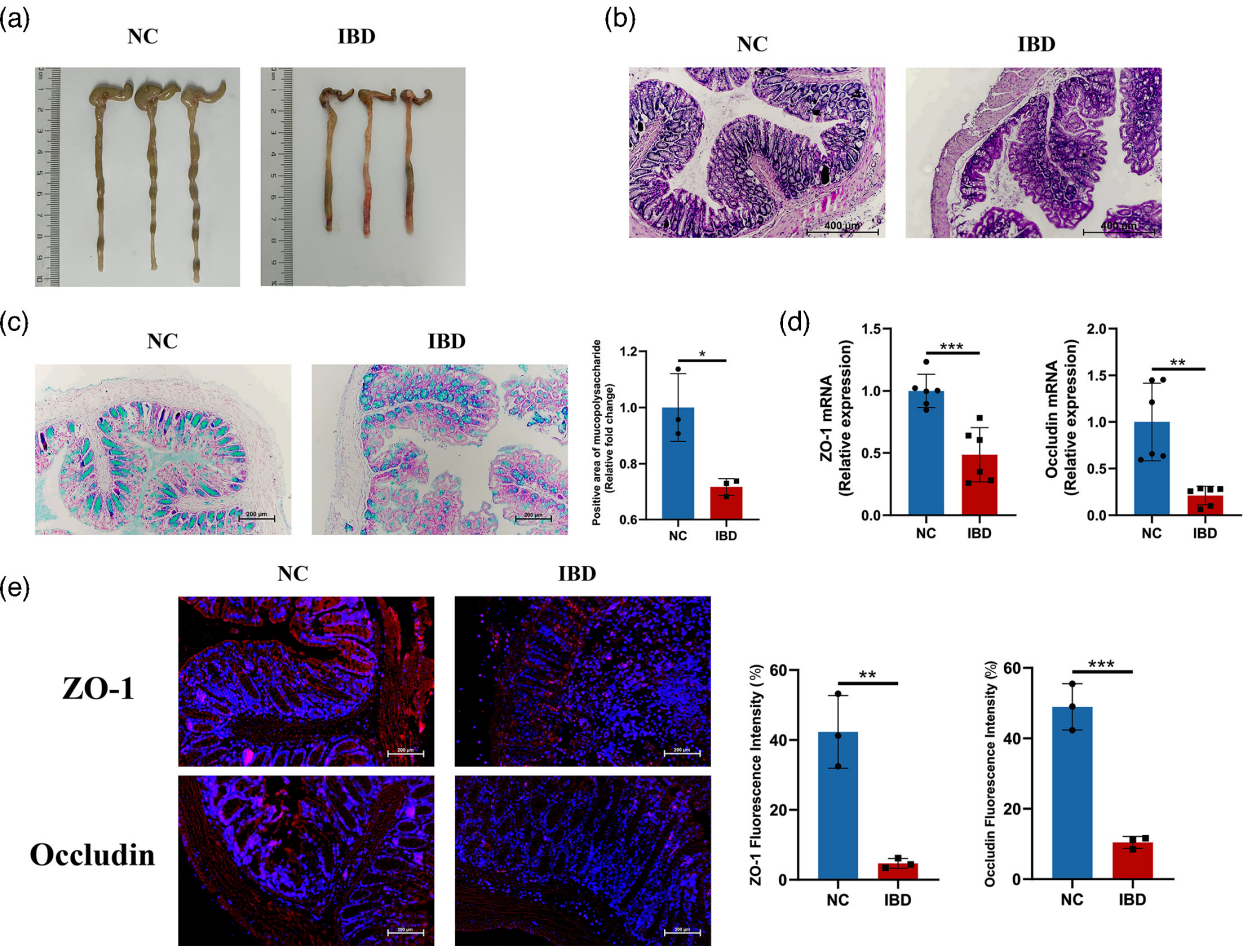
Intestinal barrier impairment and enhanced colonic permeability in IBD mice. After the mice were sacrificed, HE staining, Alcian blue staining and immunofluorescence staining were used to observe the significant differences in colonic morphology between the NC group and the IBD group. (**a**) Colon morphology; (**b**) HE-stained sections showing histopathological changes; (**c**) Alcian blue staining showing mucopolysaccharide content in goblet cells; (**d**) relative mRNA expression levels of ZO-1 and occludin in colonic tissues analysed by qRT-PCR; (**e**) immunofluorescence staining of ZO-1 and occludin proteins in colonic epithelium. Data represent mean±sem. **P<0.01; ***P<0.001.

Significant differences in colonic morphology were observed between the NC and IBD groups. In the NC group, the colonic mucosal tissue exhibited uniform colour, clear texture and regular luminal structure, with no apparent signs of congestion or oedema. In contrast, the IBD mice displayed pronounced congestion and oedema in the colonic mucosa, accompanied by a rough surface and a tendency for ulceration. The colonic lumen appeared irregular and structurally disorganized (Fig. 1a). These pathological changes indicate significant pathological damage in the colon of IBD mice.

The results of HE staining revealed that in the NC group, the colonic mucosa exhibited an intact structure, with a monolayer of columnar epithelial cells tightly arranged and uniformly eosinophilic cytoplasm. Goblet cells were clearly visible, interspersed among the epithelial cells, with mucin-filled vacuolar structures. The lamina propria showed well-organized connective tissue without significant inflammatory cell infiltration, whereas the IBD mice showed compromised epithelial integrity characterized by multiple areas of epithelial detachment and erosion. A marked reduction in goblet cell density was observed. Additionally, the lamina propria showed extensive diffuse infiltration of inflammatory cells (Fig. 1b), and the mucopolysaccharide content secreted by colonic goblet cells in the IBD mice was significantly reduced compared to the NC group (*P*<0.01) (Fig. 1c).

### Decreased expression of colonic tight junction proteins ZO-1 and occludin in IBD mice indicates increased intestinal permeability

qRT-PCR analysis revealed a significant reduction of colonic ZO-1 (*P*<0.001) and occludin (*P*<0.01) mRNA expression in the IBD group compared with the NC group (Fig. 1d). Immunofluorescence staining also revealed distinct localization patterns of these proteins. In the NC group, ZO-1 and occludin proteins showed continuous and well-defined fluorescent bands at intercellular junctions, with homogeneous fluorescence intensity; these proteins surround colon epithelial cells intact and play a key role in maintaining the tight intercellular junction structure and intestinal barrier function. However, the IBD mice exhibited discontinuous fluorescent bands and attenuated fluorescence intensity of ZO-1 and occludin proteins in the colon, with some regions showing almost complete loss of detectable fluorescence signals. Quantitative analysis revealed significantly reduced protein levels of ZO-1 (*P*<0.01) and occludin (*P*<0.001) compared to the NC group (Fig. 1e). Taken together, intestinal tight junction proteins are essential for maintaining the intestinal barrier function, and their absence can result in increased intestinal permeability and disruption of intestinal barrier function.

### Tissue distribution analysis of GFP-*E. coli*

**Fig. 2.**
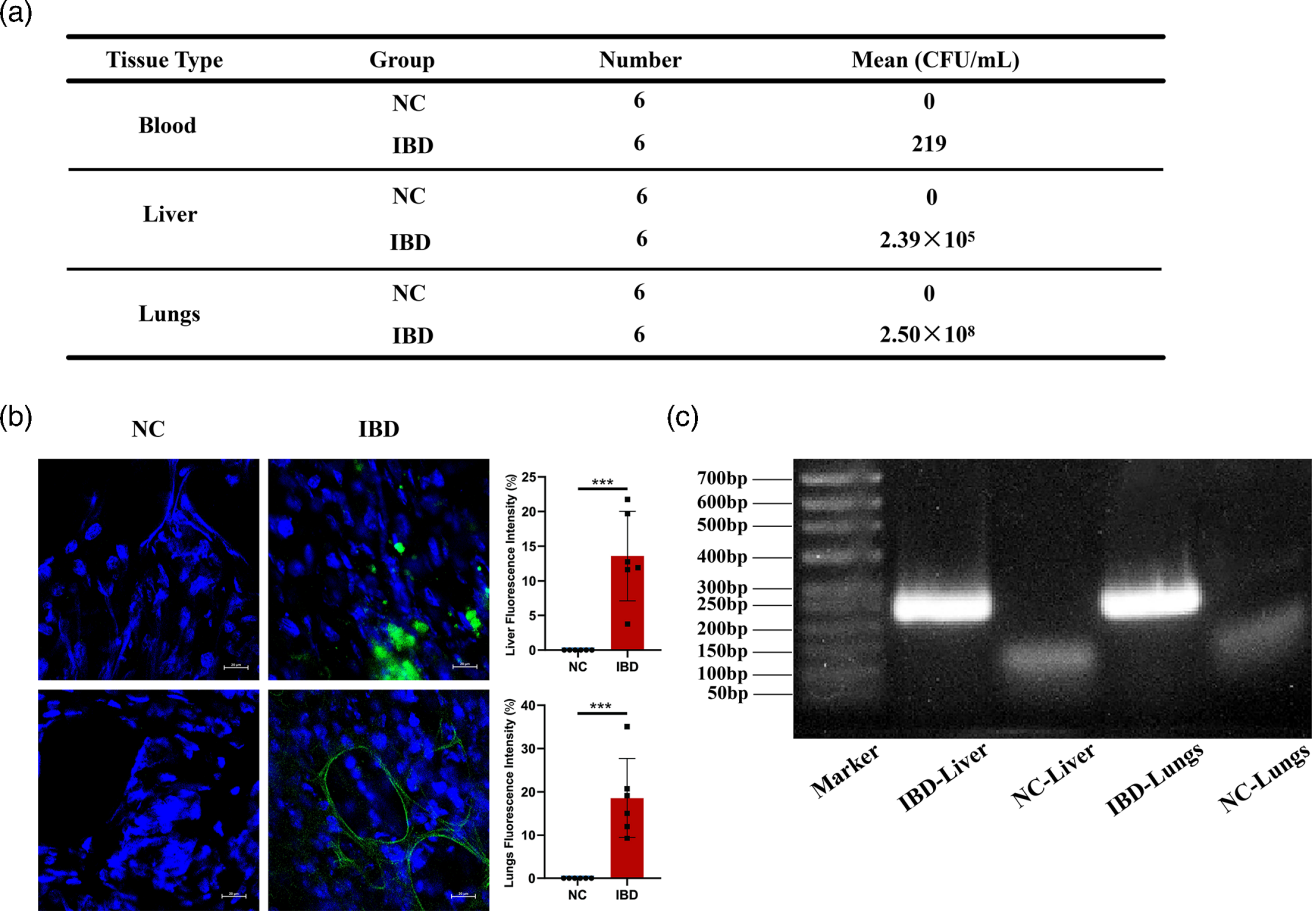
Translocation of GFP-*E. coli* from the gut to liver and lungs in IBD mice. Note: The distribution and levels of GFP-*E. coli* in liver and lungs were detected by confocal microscopy and PCR in order to observe the bacterial translocation during intestinal barrier dysfunction. (**a**) Quantification of GFP-*E. coli* colonization levels in peripheral circulation and visceral organs. (**b**) Confocal micrographs demonstrating the distribution of GFP-*E. coli* in liver and lung tissues. (**c**) Validation of bacterial dissemination in liver and lung tissues through *E. coli*-specific PCR. Data represent mean±sem. ***P<0.001.

To determine bacterial translocation pathway following intestinal barrier dysfunction, the blood and tissue homogenates (heart, liver, spleen, lungs, kidneys and oviducts) from healthy controls and IBD model mice were plated on ampicillin-containing agar plates for bacterial culture. In control mice, no bacterial colonization was detected in any of the tissues examined. In contrast, IBD mice showed systemic bacterial translocation with GFP-*E. coli* colonies identified in blood (219 c.f.u. ml^−1^), liver (2.39×10^5^ c.f.u. ml^−1^) and lung tissues (2.50×10^8^ c.f.u. ml^−1^) (Fig. 2a). The colonization hierarchy followed a descending gradient: lungs>liver>blood, and no GFP-*E. coli* colonies were detected in the heart, spleen, kidneys and oviducts (Fig. S1, available in the online Supplementary Material), suggesting that impaired gut barrier function facilitates bacterial haematogenous spread and multi-organ invasion.

### Translocation of GFP-*E. coli* from the gut to the liver and lungs via the bloodstream in IBD mice

Confocal microscopy analysis showed that no GFP-*E. coli* colonization was detected in the liver and lung tissues of the control mice, whereas IBD mice exhibited bacterial colonization characterized by the following: (1) alveolar adhesion: oval-shaped bacterial aggregates adhering to alveolar walls; and (2) hepatic infiltration: polymorphic clusters within the interstitial spaces of the liver. Quantitative analysis revealed significantly higher bacterial loads in IBD mice compared to controls (*P*<0.001) (Fig. 2b). Molecular analysis by *E. coli*-specific PCR amplification showed no detectable target bands in liver and lung samples from healthy controls. In contrast, IBD mice exhibited distinct bands corresponding to *E. coli* genomic markers in both liver and lung tissues, confirming bacteraemic dissemination in IBD mice (Fig. 2c).

## Discussion

The distribution of micro-organisms in healthy organisms remains a subject of ongoing debate. Early research predominantly supported the idea that organs are sterile. Qin *et al.* [7] developed a model that suggested a ‘pure’ internal environment in healthy individuals. Based on the technological capabilities of the time, he suggested that vital organs – including the heart and liver – were devoid of microbial life. However, advances in detection methods have challenged this paradigm. Acharya and Bajaj [2] and Chen *et al.* [8] used highly sensitive techniques to identify distinct microbial communities residing in organs such as the liver and lungs. These findings have fundamentally overturned the traditional view of organ sterility and reshaped our understanding of microbial distribution in healthy organisms.

**Fig. 3.**
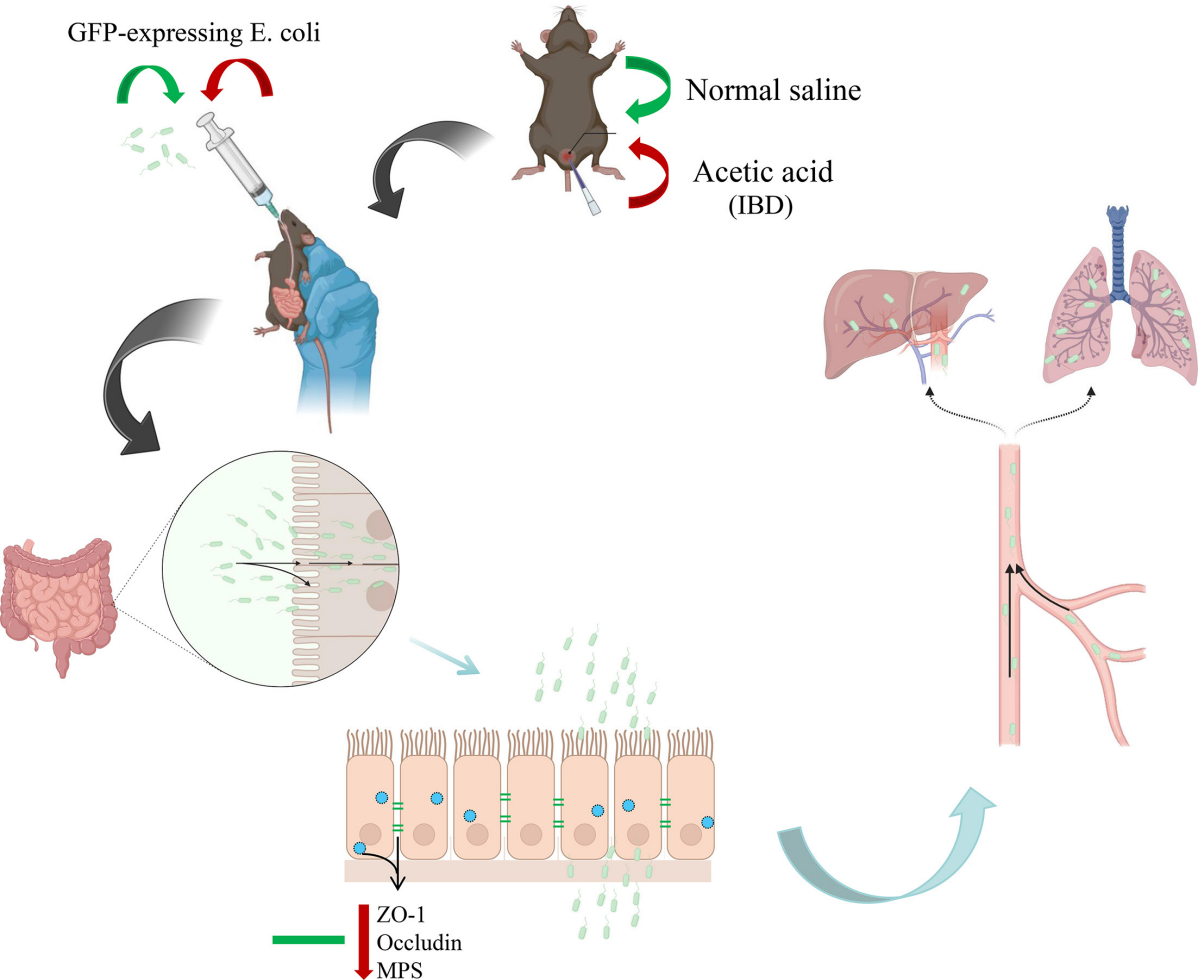
The mechanism of intestinal barrier dysfunction leading to microbial translocation. Note: The mice were randomly divided into NC and IBD groups. The mice were given either a normal saline enema (NC) or a glacial acetic acid enema (IBD). Both groups were then given GFP-*E. coli* by gavage. The results showed that there was no change in the expression of colonic mucopolysaccharides and tight junction proteins ZO-1 and occludin in the NC group, while the expression of colonic mucopolysaccharides and tight junction proteins ZO-1 and occludin was significantly reduced in the IBD group, which led to an increase in the permeability of the colon, and the GFP-*E. coli* in the IBD mice was translocated through the colon and reached the liver and lungs via the blood circulation.

In this study, we systematically tracked the distribution of GFP-*E. coli* in mice and compared microbial translocation patterns between healthy individuals and those with induced intestinal barrier dysfunction. The results showed that GFP-*E. coli* remained exclusively localized to the intestinal lumen in control mice, with no detectable colonization in systemic tissues – including blood, heart, liver, spleen, lungs, kidneys or reproductive organs. This is consistent with established theories of intact intestinal barrier function under physiological conditions [9]. In contrast, mice with experimentally induced leaky gut exhibited significant GFP-*E. coli* translocation, with increased bacterial loads detected in blood, liver and lungs. These findings demonstrate that IBD-associated barrier dysfunction increases intestinal permeability, facilitating bacterial migration from the gut lumen to extraintestinal organs (Fig. 3). This mechanistic link between intestinal barrier dysfunction and microbial translocation supports existing models of gut-derived bacteraemia [10].

GFP-*E. coli* provided a visual demonstration of the process by which *E. coli* breaches intestinal defences and progressively invades distant organs via the circulatory system. In mice with induced intestinal barrier dysfunction (leaky gut), bacteria and their by-products can travel through the portal vein to the liver, disrupting its relatively stable microecological and metabolic environment [11]. Furthermore, Dickson *et al.* [12] reported that gut-derived micro-organisms can breach the respiratory mucosal barrier and migrate to the lungs. These findings underscore the significance of our study in elucidating bacterial translocation pathways and provide deeper insights into the gut–liver axis and gut–lung axis, supporting the hypothesis that gut dysbiosis influences systemic inflammatory networks, aligning with Mousa *et al.* [13] proposal of a link between gut dysbiosis and systemic diseases.

Current research on bacterial translocation following intestinal barrier disruption is predominantly focused on the gut microbiota at a community level [14]. These studies aim to elucidate how structural and compositional changes in microbial populations correlate with pathogen dissemination during barrier compromise. While our investigation adopts a targeted approach, using GFP-*E. coli* to map precise translocation routes and unravel the mechanistic basis of bacterial pathogenicity. Traditional methods for detecting bacterial translocation – such as culture-based assays and PCR – have critical limitations. These techniques provide only static, end-point data with limited spatio-temporal resolution, limiting their ability to capture dynamic dissemination processes. The GFP-tracking system overcomes these limitations by enabling real-time visualization of bacterial migration, in line with emerging paradigms for spatial microbial dynamics proposed by Chen *et al*. [15].

While this work provides novel insights into *E. coli* translocation dynamics, several limitations warrant consideration. First, although GFP labelling enabled real-time visualization of bacterial migration, resolving the precise spatio-temporal details of translocation events (e.g. subcellular interactions or transient vascular adherence) requires integration with advanced imaging modalities. Super-resolution microscopy, high-speed live imaging and single-cell tracking systems could refine our understanding of critical translocation timelines and organ-specific invasion routes [16]. Additionally, our focus on *E. coli* as a model organism inherently overlooks the complexity of gut microbiota interactions. *In vivo*, bacterial translocation is likely to involve synergistic or antagonistic crosstalk between microbial communities and dynamics. For instance, shifts in commensal populations may either facilitate or inhibit the spread of *E. coli*. Therefore, it is difficult to comprehensively uncover the intrinsic relevance of bacterial translocation by studying only a single bacterium, which is contrary to the view of the overall role of the bacterial community [17]. Finally, the acetic acid-induced mouse model of IBD differs substantially from human IBD in both pathogenesis and pathological manifestations, lacking the chronic progression, multifactorial complexity and patient-specific heterogeneity characteristic of clinical cases. Consequently, integrating complementary approaches – including genetically engineered models and microbiota-based systems – provides a more physiologically relevant framework for studying human IBD pathophysiology.

In summary, our findings demonstrate that healthy mice exhibit no systemic colonization by *E. coli*, whereas mice with intestinal barrier dysfunction (leaky gut) show significant bacterial translocation to the bloodstream, liver and lungs. This provides vital insights into how leaky gut facilitates microbial dissemination. By visualizing spatio-temporal dynamics of *E. coli* migration via GFP tracking, current research continues to elucidate the gut–liver and gut–lung axes, demonstrating how intestinal damage drives multi-organ dysfunction in IBD. These findings align with known associations between gut dysbiosis and systemic pathologies [13]. Future studies integrating multi-species models and advanced imaging technologies may further unravel the complex interplay between gut integrity, microbial communities and systemic health.

## Supplementary material

10.1099/jmm.0.002054 Uncited Fig. S1.

## Abbreviations

GFP-*E. coli*: GFP-labelled *Escherichia coli*
IBD: inflammatory bowel disease
NC: normal control
qRT-PCR: quantitative reverse transcription PCR
