# Supplementary material for: Exploration of the distribution of intestinal bacteria in mice under normal and intestinal leakage (IBD) conditions
Source: J Med Microbiol. 2025 Sep 3;74(9):002054. doi: 10.1099/jmm.0.002054 (PMC12408187; doi:10.1099/jmm.0.002054)
Supplement: Uncited Fig. S1. [file jmm-74-02054-s001.pdf]

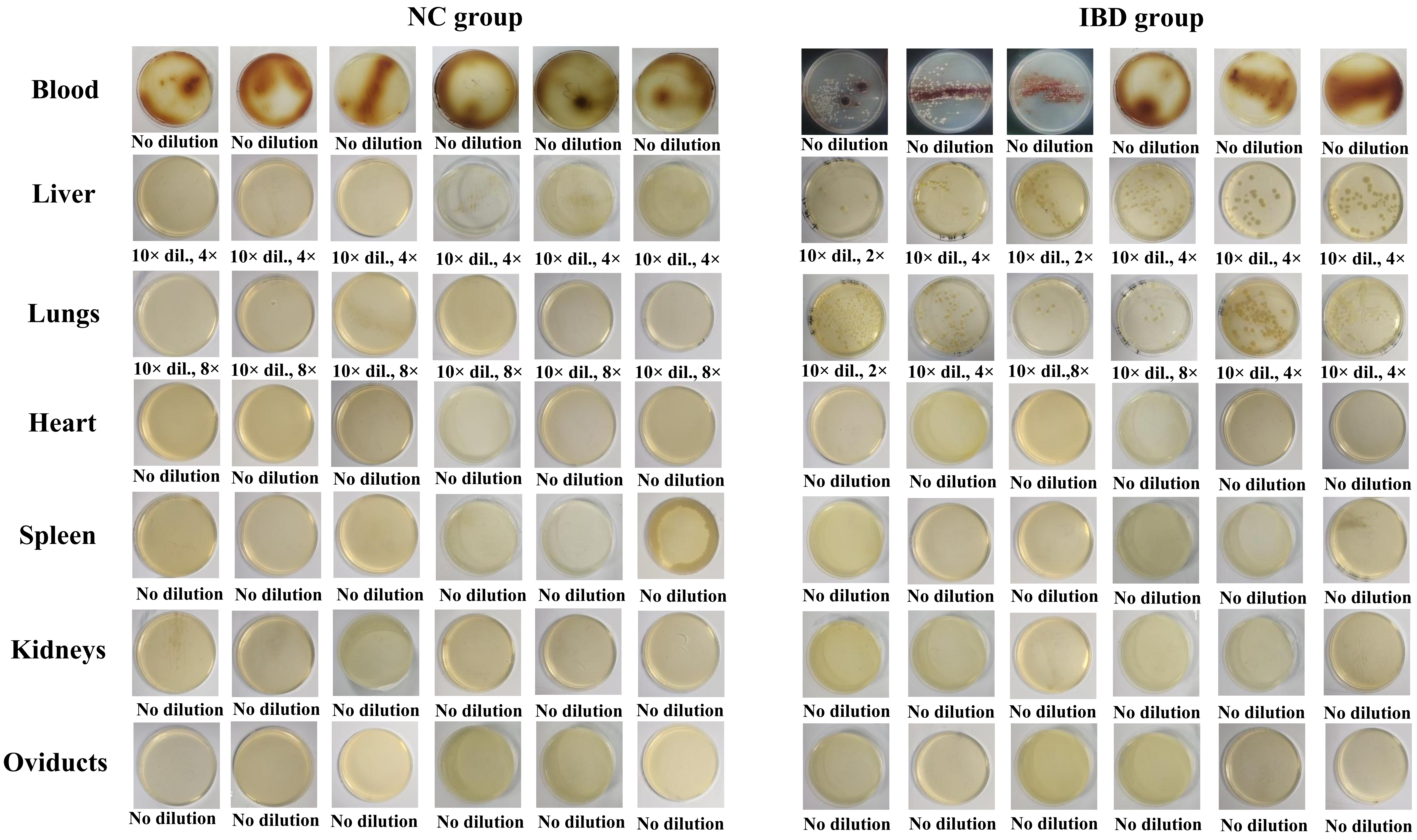

Supplementary Figure 1. GFP-E. coli colonies in various tissues of NC and IBD mice.

Note: To detect the distribution of GFP-E. coli in various tissues, the blood, liver, lungs, heart, spleen, kidneys, and oviducts were collected from the mice. The liver tissue homogenate was serially diluted 10-fold four times (abbreviated as “10× dil., 4×” in the figure); the lung tissue homogenate was serially diluted 10-fold eight times (abbreviated as “10× dil., 8×” in the figure), while the blood and other tissues were not diluted. The blood and each tissue homogenate were plated onto ampicillin-containing Petri dishes, incubated at 37°C for 24 hours, and the colony number was used to quantify GFP-E. coli.
